# Generic Health Utility Measures in Exercise Oncology: A Scoping Review and Future Directions

**DOI:** 10.3390/curroncol30100642

**Published:** 2023-09-28

**Authors:** Joanna F. Parkinson, Paula A. Ospina, Jeff Round, Margaret L. McNeely, C. Allyson Jones

**Affiliations:** 1 Department of Physical Therapy, Faculty of Rehabilitation Medicine, University of Alberta, Edmonton, AB T6G 2G4, Canada; jfparkin@ualberta.ca (J.F.P.); pospina@ualberta.ca (P.A.O.); cajones@ualberta.ca (C.A.J.); 2 Department of Pediatrics, Faculty of Medicine and Dentistry, University of Alberta, Edmonton, AB T6G 1C9, Canada; jround@ihe.ca; 3 Institute of Health Economics, Edmonton, AB T5J 3N4, Canada; 4 Department of Oncology, Cancer Care Alberta, Edmonton, AB T6G 1Z2, Canada; 5 School of Public Health, University of Alberta, Edmonton, AB T6G 1C9, Canada

**Keywords:** health utility measures, exercise oncology, cost-effectiveness, scoping review

## Abstract

Despite the evidence that exercise is effective at mitigating common side effects in adults with cancer, it is rarely part of usual cancer care. One reason for this is the lack of economic evidence supporting the benefit of exercise. Economic evaluations often rely on the use of generic utility measures to assess cost effectiveness. This review identifies and synthesizes the literature on the use of generic utility measures used to evaluate exercise interventions for adults with cancer. A systematic search of the literature from January 2000 to February 2023 was conducted using four databases (Medline, EMBASE, CINAHL, Academic Search Complete). Exercise studies involving adults with any type of cancer that used a generic utility measure were eligible for inclusion. Of the 2780 citations retrieved, 10 articles were included in this review. Seven articles included economic evaluations, with varying results. Four studies reported on cost-effectiveness; however, detailed effectiveness data derived from the generic utility measure were often not reported. Generic utility measures help to compare baseline values of and changes in health utility weights across studies and to general population norms; however, to date, they are underutilized in exercise oncology studies. Consideration should be given to the identified research evidence, population, and methodological gaps.

## 1. Introduction

Exercise is an evidence-based strategy to address many of the negative effects of cancer treatment, including fatigue, depression, and anxiety [1,2,3]. Systematic reviews and meta-analyses show favourable effects of exercise on health-related quality of life (HRQL), according to cancer-specific measures such as the European Organization for Research and Treatment of Cancer Quality of Life questionnaire (EORTC QLQ-C30) [4] and the Functional Assessment of Cancer Therapy (FACT) [5]. Significant improvements have been found with exercise compared to control for overall HRQL [6,7,8,9,10] and specific domains including physical functioning [6,7,9,10], role functioning [7,9], emotional functioning [7], and social functioning [9]. Despite many benefits, exercise programs are often not a part of standard cancer care. Given the benefits and relatively low costs of implementing exercise programs, there is a need to move exercise oncology research from a focus on efficacy to effectiveness—meaning determining how to best implement exercise programs within standard care.

Economic evaluations provide necessary information for implementation, as policy and decision-makers often must make decisions regarding how to best allocate scarce healthcare resources [11]. Cost-effectiveness analyses (CEAs) of healthcare interventions compare the resources consumed (costs) with the health changes (consequences) resulting from the intervention (See Table 1: key terms) [12]. To best inform resource decisions, equitable comparisons across different healthcare systems are needed. Quality-adjusted life years (QALYs) is a commonly used summary measure for economic evaluations of healthcare [13]. It includes the concept of duration and health-related quality of life and is the product of the duration of time spent in a certain health state and the utility score (HRQL weight). It is expressed as a single index, which permits comparisons across different populations and conditions [11]. For instance, a QALY measurement can be obtained with an exercise program (treatment) compared with no treatment. When QALYs are the outcome of an economic evaluation, it is referred to as a cost–utility analysis (CUA) [14].

Utilities are needed to generate a QALY measurement value. Utilities are preference weights which are measured using a cardinal scale of 0–1, using anchors of 0 equivalent to being dead and 1 equivalent to full heath [14]. Negative values represent states ‘worse than death’. The measurement of health utilities can be obtained by either direct or indirect elicitation methods [15]. Methods of valuing HRQL weights using direct elicitation commonly include visual analogue scale (VAS), standard gamble (SG), or time trade-off methods (TTO) [16]; however, this type of method can be challenging for participants and is very time consuming. Indirect elicitation methods use a generic utility measure, which includes a health status classification system with pre-defined preference weights assigned to each health state [12]. Generic utility measures often include peripheral dimensions of health that are not central to the specific condition, which in this case is cancer. A range of generic measures exist with differing dimensions, levels for each dimension, and populations used as a base for the preferences. The valuation methods to derive the preferences also differ. For instance, the EQ-5D uses a TTO whereas the Health Utilities Index (HUI) and SF-6D use SG methods [17,18].

When comparing an intervention with a control or comparison intervention, an economic value can be derived using a CUA. The incremental cost-effectiveness ratio (ICER) is the ratio of the difference in cost between the intervention and comparison and the difference in effectiveness between the two groups. It summarizes the cost per unit of health benefit gained and can guide funding decisions regarding interventions [12]. Guidelines for economic evaluations from both the National Institute for Health and Care Excellence (NICE) in the United Kingdom [16] and the Canadian Agency of Drug and Technologies in Health (CADTH) [17] recommend the use of generic health utility measures for economic evaluation of healthcare interventions.

Generic utility measures have been used in cancer populations [18,19,20], but it is unknown how often these measures are used in exercise oncology studies. The purpose of this scoping review is to identify and synthesize the literature on generic utility measures used to evaluate exercise interventions for adults with cancer. Specific objectives are (1) to explore the type, frequency, and findings related to the use of utility measures in exercise oncology research; (2) to describe the study designs, characteristics of adult cancer populations, exercise prescription factors, and timing of the exercise interventions in the cancer trajectory; and (3) to identify potential research gaps in the current literature.

## 2. Materials and Methods

A scoping review based on the framework proposed by Arksey and O’Malley [21] and refined by Levac and colleagues [22] was performed to address the objectives. We also followed the PRISMA Extension for Scoping Reviews (PRISMA-ScR) [23]. A scoping review was selected rather than a systematic review as our interest was in exploring the characteristics of studies and identifying research gaps rather than providing evidence to inform clinical practice or policy [24,25]. The protocol for this review was registered on Figshare (https://figshare.com/articles/preprint/Generic_Health_Utility_Measures_in_Exercise_and_Cancer_Scoping_Review_Protocol/17868740 (accessed on 4 January 2022)).

### 2.1. Stage 1: Identifying the Research Question

Our research questions are as follows: What is the current state of the exercise oncology research using generic utility measures in adults during and after cancer treatment? Specifically, we want to know what patient populations are included, which exercise intervention parameters are prescribed, and what health utility measures are used? Furthermore, what specific metrics are reported, what are the baseline utility values, and what are the changes in utility scores? For this review, we defined exercise as “planned, structured, and repetitive bodily movement performed to improve or maintain one or more components of physical fitness” [26].

### 2.2. Stage 2: Identifying Relevant Studies

A health sciences librarian in conjunction with the research team developed search strategies for four electronic databases (Medline, Embase, CINAHL, and Academic Search Complete). Articles were limited to the English language and were published between January 2000 and February 2023. We limited the search to 2000 onwards to reflect the most current research available given advances in oncologic treatments and improved overall cancer survival [27].

Study eligibility included (1) adults (18+ years) with any type of cancer diagnosis, (2) structured physical exercise intervention that targeted multiple muscle groups and one or more health related components of physical fitness (cardiorespiratory endurance, muscular endurance, muscular strength, body composition, and flexibility) and was implemented by a qualified exercise or rehabilitation professional, (3) delivered in a group or individual format during or after cancer treatment, (4) randomized controlled trials, intervention studies, comparative studies, follow-up studies, or economic evaluations of any of the aforementioned study designs, (5) a minimum of 20 participants in the intervention group, and (6) any version of a generic utility measure as a primary or secondary outcome including EQ-5D, the Short-Form Six-Dimension (SF-6D), the Health Utilities Index Mark 2 (HUI2) and Mark 3 (HUI3), Assessment of Quality of Life (AQoL), Quality of Well-Being (QWB), and 15D©.

Studies were excluded if they were recreational activities such as yoga, dance, Pilates, tai-chi, qigong, or sport-based. Multimodal interventions, such as combined exercise and nutrition, were excluded. Interventions that included additional non-exercise therapeutic modalities such as ultrasound were also excluded. Articles were excluded if the study sample included children, adolescents, or adult survivors of childhood cancer.

### 2.3. Stage 3: Study Selection

Citations were uploaded to Covidence systematic review software version 2.0 (Veritas Health Innovation, Melbourne, VIC, Australia) for citation management and the screening process. Duplicate citations were identified and removed. Two reviewers (JFP and PAO) independently screened the titles and abstracts. “Strong” [28] inter-rater reliability (kappa = 0.9) between the 2 reviewers was reported for the first 50 citations. Disagreement between reviewers was resolved through discussion, and when necessary, through third party adjudication (CAJ, MLM). Both reviewers then independently screened half of the remaining citations. Two reviewers (JFP and PAO) independently screened the first 10 full texts with “perfect” [28] inter-rater reliability (kappa = 1.0) before each screened half of the remaining articles.

### 2.4. Stage 4: Charting the Data

Data were extracted from the full texts of the included studies. A standardized form was used to collect data on the study characteristics (author, publication year, country of publication, study design), study population characteristics (participant demographic and medical characteristics, sample size), intervention and comparators (e.g., description, duration of treatment, adherence, losses to follow-up), outcome measures, type of economic evaluation, and results. Extraction data were downloaded to Microsoft Excel for review. One author (JFP) extracted the data and two other authors (PAO, MLM) checked the data to ensure accuracy. When necessary, previous trial publications, including protocols, were accessed to extract further details about the intervention and participants.

### 2.5. Stage 5: Collating, Summarizing, and Reporting Results

To provide a broad overview of the included studies, we summarized and collated data on the cancer type, participant characteristics, exercise intervention details (frequency, intensity, type of exercise, length of session, duration of intervention), whether the intervention took place during or after cancer treatment, and the generic utility measures used, rationale for inclusion of the measure(s), and findings related to use of these measures including utility scores, QALYs, and ICERs.

## 3. Results

**Figure 1:**
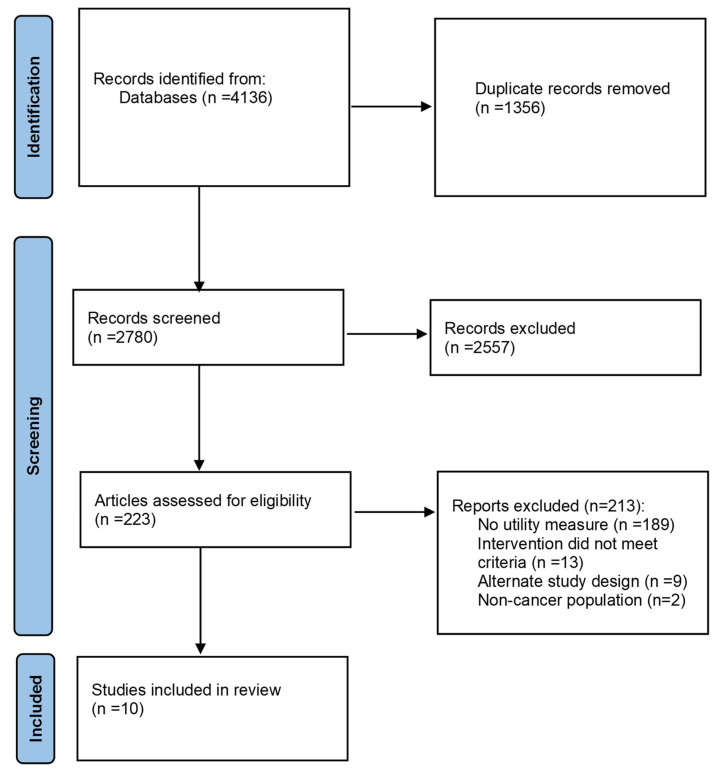
PRISMA flow diagram.

The search yielded 4136 citations of which 1356 duplicates were removed, and the remaining 2780 (67%) citations were reviewed for eligibility. In total, 223 articles were included in the full-text screen, of which 10 articles with a total of 1285 adults with cancer were included in the review. During full-text screening, the most common reason for study exclusion was not including a generic utility measure as an outcome (Figure 1).

### 3.1. Study Characteristics

The majority (60%) of the included studies were from Europe (Netherlands [29,30,31,32]; Spain [33,34]). Three studies were from Australia [35,36,37] and one was from Japan [38]. All included articles were published between 2010 and 2023 and included data collected between 2006 and 2020 (Supplementary Table S1).

Seven studies included a CUA examining incremental cost per QALY gained and included a generic utility measure to calculate QALYs [29,30,31,32,35,36,37]. Three studies included a generic utility measure but did not report a CUA, including one RCT from Japan [38], and two non-randomized studies from Spain [33,34].

### 3.2. Participants

Overall, 72% (n = 913) of participants in the ten included studies were individuals with breast cancer, with the majority of participants across studies being female (80%). The mean age reported in the 10 studies ranged from 48 to 76.2 years. Five studies were specific to breast cancer [32,33,36,37,38]. A large RCT study included 204 individuals with breast and 29 with colon cancer; however, the colon subset was relatively small (n = 14 in the intervention and n = 15 in the control) [30]. Another RCT study of 277 participants included those diagnosed with breast cancer (n = 181), colon cancer (n = 49), lymphomas (n = 26), ovarian (n = 12), testis (n = 5), and cervix cancer (n = 4) [29]. The remaining three studies were specific to prostate cancer (n = 100) [35], lung cancer (n = 34) [34], and hematological cancers (n = 109) [31].

### 3.3. Exercise Interventions

All of the exercise interventions included combined aerobic and resistance exercise training, with four taking place during cancer treatment [30,32,34,37] and five after treatment [29,31,33,35,38]. One intervention took place after breast cancer surgery, with the majority of participants receiving at least one type of treatment (chemotherapy, radiotherapy, and/or hormone therapy) during the intervention, but being on treatment was not a requirement to participate [36]. All exercise sessions were 45 to 60 min in length, occurring from one to three times a week over 8-week to 8-month periods. Two interventions were home-based, with one using an app to deliver the intervention [38] and one providing participants with a DVD of the exercises [37]. One intervention included both in-person and independent home-based exercise sessions [36]. The remaining seven interventions were fully in-person.

### 3.4. Utility Measures Results

Nine studies used the EQ-5D-3L [29,30,31,32,33,34,36,37,38] and one study used the SF-6D [35]. Only five studies (50%) reported utility scores, including individuals with breast cancer (n = 579) and colon cancer (n = 29), and used the EQ-5D-3L to derive utilities [30,33,36,37,38]. Three of these studies also included CUAs [30,36,37].

Four studies found no statistically significant results (*p* > 0.05) for utility scores [30,33,37,38]. Only one RCT found a clinically meaningful and significant change (*p* = 0.037) in utility scores (+0.07) over time, favoring the intervention group (n = 127), and a clinically meaningful difference between groups compared with usual care (n = 60) during an 8-month program [36]. In this study, the authors considered a difference of ≥0.06 of the EQ-5D-3L to be clinically meaningful, which aligns with other research on the MCID of utilities [36].

Four of the remaining studies calculated utilities for the CUA but did not report the values, only the QALYs gained and ICERs. Three of these studies used the EQ-5D-3L [29,31,32] and one used the SF-5D [35]. One study, which used the EQ-5D-3L, did not include a CUA nor calculate utilities. Instead, the authors calculated an overall score by summing the score for each domain [34], which is not a validated method for scoring the EQ-5D-3L [39]. Results of all studies are summarized in Table 2.

All seven articles that included a CUA reported results for QALYs. Five of these studies found an incremental gain in QALYs with exercise compared to control [29,32,35,36]. The largest gain was 0.04 QALYs (95% CI 0.01–0.08) and was found in a study of individuals with breast cancer who exercised during chemotherapy [29,32,35,36]. The ICER for this study was EUR 26,916/QALY, which may be considered cost-effective depending on the willingness-to-pay threshold, which in the Netherlands is reported to range from EUR 20,000 to EUR 80,000 [40]. The smallest gain was 0.0085 QALYs (95% CI −0.0093–0.0256). This study included individuals with prostate cancer who exercised after treatment, and resulted in an ICER of AUD 64,235/QALY, which is unlikely to be cost-effective as it exceeds the typical Australian willingness-to-pay threshold of AUD 50,000 [35].

One study found a decrease of 0.07 QALYs (95%CI −0.17–0.04) in individuals with hematological cancers who exercised after treatment [31]. The ICER for this intervention was −8043, indicating that the intervention was more costly and less effective than usual care [31]. Another study found a decrease of 0.01 QALYs (95% CI not reported) and no change when outliers were excluded [37]. This study included individuals newly diagnosed with breast cancer and the intervention took place during treatment, and resulted in ICERs of AUD 484,884/QALY (full dataset), well above the threshold of AUD 50,000/QALY [37].

## 4. Discussion

Our review findings indicate that generic utility measures are not commonly included in exercise oncology studies. Furthermore, an evidence gap was seen in the reporting of generic utility measures in exercise oncology studies. While four studies calculated utilities for a CUA, they did not report the actual utility scores, only the QALYs and ICERs [29,31,32,35]. Although two of these studies had favourable ICERs [29,32], utility scores help to characterize the baseline health status of the study sample, and inform the magnitude and direction of change over time. Moreover, the values allow comparison across studies and can indicate whether the change was meaningful to participants. When considering the cost per QALY as the primary outcome for economic evaluations, we found contradictory and inconclusive results, which are similar to findings in systematic reviews of economic analyses in exercise oncology [41,42,43]. Similar to these reviews, we noted variability in patient characteristics, time horizons, and exercise parameters of the included studies, which probably contributed to the mixed results. Overall, these findings support the need for further research with larger sample sizes.

Our findings suggest that there is an evidence gap in our understanding of the optimal exercise type, timing, and intensity. For example, consistent with previous reports [41], higher intensity interventions show promise for being cost-effective when delivered post-treatment. This finding was supported by the study by Kampshoff and colleagues involving 277 individuals with mixed cancer types, where the exercise intervention took place following completion of chemotherapy [29]. The authors found that high intensity aerobic and strength training showed benefits for outcomes of fatigue and anxiety, and was cost-effective compared with low-moderate-intensity exercise [29]. On the other hand, a study conducted by van Dongen and colleagues examined high-intensity exercise for individuals with multiple myeloma and non-Hodgkin lymphoma who were undergoing treatment involving autologous stem cell transplantation [31]. The authors reported that high-intensity exercise did not result in significant changes in fitness nor fatigue, and was also not cost-effective when compared with usual care [31]. While the discordant findings may be explained by differences in the timing of the intervention in relation to cancer treatment (following versus during intensive treatment), the results were probably also influenced by patient characteristics (e.g., stage of cancer) and differences in completion rates between the two studies (i.e., 75% and 54%, respectively).

The setting and supervision of exercise programs may also be an important factor in determining both effectiveness and cost-effectiveness. While both supervised and unsupervised exercise have advantages and disadvantages, the optimal approach for people with cancer remains a source of debate [44,45,46,47,48,49,50]. In this review, only two unsupervised, home-based interventions were included, and conclusions cannot be made regarding the effects setting and supervision have on utility scores, QALYs, and cost-effectiveness.

Economic evaluations, given their focus on costs and treatment effects, require careful consideration of research methodology pertaining to study power and the time horizon for collection of outcome effects [13]. For example, two breast cancer-specific studies, both of which took place in the Netherlands during treatment and were similar in duration, frequency, and type of exercise, resulted in vastly different ICERs of EUR 26,916/QALY [32] and EUR 403 394/QALY [30]. This large difference in ICERs may be explained partially by the differences in the reported healthcare and societal costs between the two studies. May and colleagues reported higher costs, length of hospital stay, and sick leave compared with control participants [30], whereas, van Waart and colleagues reported a more favourable ICER, while healthcare and societal costs did not differ significantly across groups [32]. Better chemotherapy completion rates in the supervised exercise group (a finding consistent with the study by May and colleagues) led to higher chemotherapy costs. While costs were higher with exercise, better chemotherapy completion is associated with improved cancer survival outcomes [51,52,53], suggesting the need for longer-term follow-up of cancer outcomes, and the potential for underestimation of the cost-effectiveness of exercise. A recent systematic review by Wang and colleagues found that five of six (83%) studies that used decision-analytic modelling to extrapolate long-term health effects of exercise (3 years to lifetime) were cost-effective, whereas only five of ten (50%) trial-based analyses were cost-effective. Time horizons for the trial-based analyses ranged from 9 to 16 months [43].

Another important finding was related to limitations inherent in the chosen health utility measures, namely, the reported ceiling effects and poor sensitivity to change associated with the EQ-5D-3L, a health utility measure that was used in nine of the ten studies in this review [36]. The EQ-5D-5L has been shown to have increased sensitivity and precision over the 3L version, and is recommended for future work [54]. Moreover, unlike condition-specific measures, generic utility measures often do not assess the central domains of HRQL for a specific disease such as cancer. For example, the EQ-5D does not have a measure of energy or fatigue, which is a commonly reported symptom that is important to adults with cancer [55]. The generic utility measures may not be as responsive to change as a condition-specific measure; however, they can complement their use by providing a multi-dimensional construct that allows comparison of cost-effectiveness across interventions and disease conditions. Thus, generic utility measures are important to facilitate economic evaluations of exercise oncology programs, but are most informative when findings are considered in addition to, not instead of, cancer-specific HRQL measures.

Studies in our review largely involved individuals with breast cancer. This finding is not surprising, given that a majority of research in the exercise oncology field has focused on women with breast cancer [3]. However, this population gap limits the generalizability of our results to other cancer types. Moreover, most of the studies included in this review were supervised, in-person interventions. Only one study was found that used a health application to deliver the exercise intervention. Future studies involving the use of technology should consider inclusion of generic utility measures to inform cost-effectiveness. Given the heterogeneity in patient characteristics, timing of exercise interventions and exercise programming features, more large-scale studies are warranted, especially in cancers other than breast cancer.

## 5. Conclusions

**Figure 2:**
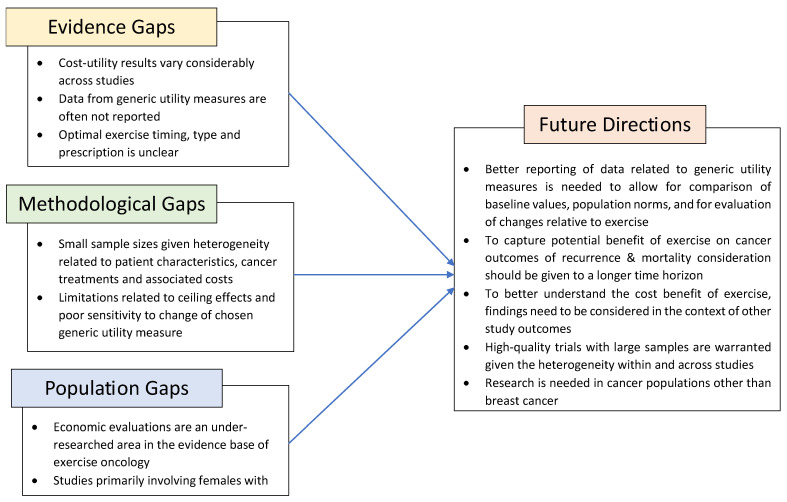
Identified research gaps and future considerations in exercise oncology research.

Generic utility measures are important to inform economic evaluations; however, to date, they have been underutilized in exercise oncology studies. We identified research gaps relative to evidence, methodology, and population (Figure 2). To provide more rigorous economic evaluations of exercise in oncology, researchers should report utility scores when conducting CUAs, in addition to QALYs and ICERs. Findings related to utility scores should be considered alongside other key metrics including the impact of exercise on cancer-related symptoms, fitness, and quality-of-life outcomes. Despite the limited evidence of cost effectiveness, the established evidence supporting the benefit of exercise for health-related quality of life, physical functioning, fatigue, anxiety, and depression supports consideration for inclusion in standard care [1,2,3].

## Figures and Tables

**Table 1:** Economic Evaluations: Key Terms.

Term	Definition
Health economic evaluation	Investigation of the value for money of different health interventions. Information is used to inform a recommendation for adoption of a new treatment in routine practice. There are four main types of health economic evaluations: (i) cost-minimization, (ii) cost-effectiveness analyses, (iii) cost-utility analyses, and (iv) cost-benefit analyses [11].
i. Cost minimization	This analysis is used when the outcome or benefit of the intervention is the same, and the costs are simply compared [11].
ii. Cost-effectiveness analyses (CEA)	Comparative analysis of the costs and outcomes (cost-effectiveness ratio) of two or more intervention alternatives with a common health outcome measured in natural units (i.e., life years gained, disease case averted). Usually tested using a randomized controlled trial design [12].
iii. Cost–utility analyses (CUA)	Comparative analysis of two or more different health intervention alternatives with different health outcome measures. Allows consideration of multiple outcomes (i.e., benefit for fitness and symptoms). Effects are measured through quality-adjusted life years (QALYs) [13].
iv. Cost–benefit analyses	A complex form of analysis that compares the costs of two or more intervention alternatives in terms of their relative benefit on direct, indirect, and intangible costs based on preferences of those affected (willingness to pay or loss/gain in income due to illness) [13].
Time horizon	Period over which health outcomes/effect data and costs are collected [14].
Quality-adjusted life years (QALYs)	QALYs capture the quantity and quality of life years in a single measure of health outcome [14]. The individual’s health is assessed using a preference-based quality of life measure; and the value is converted into a health utility value (i.e., a common currency). Calculation of QALY = an individual’s utility values are multiplied by the time that is spent in specific health state (i.e., length of time or life years saved adjusted for any loss in quality of life) [12].
Utility	Utility is a measure to reveal preferences for a given health state, ranging from 0 (death) to 1 (full health) [13].
Time trade-off method	A direct method of determining the health utility state where the choice is between living the rest of life in an impaired state, or living in full health for a shorter period of time [15].
Standard gamble methods	A direct method of determining the health utility state where the choice is between the certainty of remaining in a particular health state or taking a gamble of either being in full health or risking death. The probability of experiencing death varies until the individual is indifferent between the certainty and the gamble [15].
Generic utility measure	Generic utility measures are health-related quality-of-life instruments that are used as an indirect method of estimating utility values for computing QALYS. Commonly used generic utility measures include the EuroQol (EQ-5D), Short Form (SF-6D), and the Health Utilities Index (HUI) [13]. Valuation methods used may include the time trade-off (i.e., EQ-5D) and standard gamble methods (i.e., SF-6D and HUI).
Incremental cost-effectiveness ratio (ICER)	The ratio of the difference in cost between interventions (e.g., exercise versus control) and the difference in benefit between the two interventions. Interventions that show improved benefit and are less costly are more likely to be implemented [12].

**Table 2:** Study characteristics and findings.

Study/Country	Study Design	Reason for Including Generic Utility Measure	Measure Used/Timing of Measurement	Main Findings			
				EQ-VAS Scores	Utility Scores	QALYs	ICERs
Breast Cancer Only							
Gordon et al., 2017 [36], Australia	Cost–utility/cost-effectiveness analysis of an RCT	To calculate QALYs for the economic evaluation	EQ-5D-3L Baseline (6 weeks post-surgery), 6 months post-surgery, 12 months post-surgery	Not reported	Intervention: 0.79 (BL), 0.83 (6 months), 0.86 (12 months) Control: 0.83 (BL), 0.81 (6 months), 0.85 (12 months) Clinically important within-group change in intervention group from baseline to 12 months *p*-value: 0.037 *	Incremental gain in exercise group was 0.009 QALYs (95% CI not reported)	Model 1 (service provider model): AUD 105 231 and model 2 (private model): AUD 90 842
van Waart et al., 2018 [32], The Netherlands	Cost–utility/cost-effectiveness analysis of an RCT	To calculate QALYs for the economic evaluation	EQ-5D-3L Baseline, every 3 months during chemo, end of chemo, 3- and 6-months post chemo	Not reported	Not reported	Incremental gain in exercise group was 0.04 QALYs (95% CI 0.01–0.08)	Exercise versus UC was EUR 26,916/QALY
Haines et al., 2010 [37], Australia	RCT with cost–utility/cost-effectiveness analysis	To evaluate both efficacy and economic efficiency	EQ-5D-3L Baseline, 3 months, 6 months	Intervention: 72.6 (BL), 80.6 (3 months), 80.4 (6 months) Control: 77.5 (BL), 74.1 (3 months), 79.3 (6 months) *p*-value: 0.09 *	Intervention: 0.81 (BL), 0.78 (3 months), 0.80 (6 months) Control: 0.85 (BL), 0.84 (3 months), 0.83 (6 months) *p*-value: 0.87	QALYs were -0.01 (full dataset) and 0 (outliers excluded) (95% CI’s not reported)	AUD 484,884/QALY (full dataset) or AUD 340,391/QALY (outliers excluded)
May et al., 2017 [30], The Netherlands (Breast cancer subgroup)	Cost–utility/cost-effectiveness analysis of an RCT	To calculate utilities and QALYs for the economic evaluation	EQ-5D-3L Every 4 weeks for 36 weeks	Not reported	Intervention: 0.88 (BL), 0.82 (36 weeks) Control: 0.87 (BL), 0.82 (36 weeks)	Incremental gain in exercise group was 0.01 QALYs (95% CI −0.02–0.03)	EUR 403 394/QALY
Ochi et al., 2022 [38], Japan	RCT	To measure HRQL	EQ-5D-3L Baseline, 12 weeks	Not reported	Intervention: 0.95 (BL), 0.92 (12 weeks) Control: 0.94 (BL), 0.88 (12 weeks) *p*-value: 0.25	Not reported	Not reported
Cuesta-Vargas et al., 2014, [33] Spain	Non-randomized controlled intervention study	To measure quality of life	EQ-5D-3L Baseline and 8 weeks	Intervention: 28.3 (BL) 49.6 (8 weeks) Control: 29.3 (BL), 32.5 (8 weeks) *p*-value: 0.001 *	Intervention: 0.29 (BL), 0.32 (8 weeks) Control: 0.28 (BL), 0.33 (8 weeks) *p*-value: 0.068	Not reported	Not reported
Other Cancers							
Kampshoff et al., 2018 [29], The Netherlands	RCT with cost–utility/cost-effectiveness analysis	To calculate QALYs for the economic evaluation	EQ-5D-3L Baseline, 12 weeks, 64 weeks	Not reported	Not reported	Incremental gain in exercise group was 0.028 QALYs (95% CI −0.006–0.061)	Cost savings of EUR 87,831 per QALY gained for high-intensity exercise compared with low intensity exercise
May et al., 2017 [30], The Netherlands (Colon subgroup)	Cost–utility/cost-effectiveness analysis of an RCT	To calculate utilities and QALYs for the economic evaluation	EQ-5D-3L Every 4 weeks for 36 weeks	Not reported	Intervention: 0.89 (BL), 0.89 (36 weeks) Control: 0.82 (BL), 0.79 (36 weeks)	Incremental effect was 0.03 QALYs	Cost-savings of EUR 4321/QALY
van Dongen et al., 2019 [31], The Netherlands	RCT with cost–utility/cost-effectiveness analysis	To calculate QALYs for the economic evaluation	EQ-5D-3L Baseline, post-intervention, 1 year after PI assessment	Not reported	Not reported	Incremental change in exercise group was −0.07 QALYs (95%CI −0.17–0.04)	−EUR 8043, indicating that the intervention was more costly and less effective than usual care
Edmunds at al., 2020 [35], Australia	Cost–utility/cost-effectiveness analysis of an RCT	To calculate QALYs for the economic evaluation	SF-6D Baseline, 6 months, 12 months	Not applicable	Not reported	Incremental gain in exercise group was 0.0085 QALYs (95% CI −0.0093-0.0256)	AUD 64,235/QALY
Rosero et al., 2020 [34], Spain	Non-randomized controlled intervention study	To measure self-perceived physical function and health status/health-related quality of life	EQ-5D-3L Baseline and 10 weeks	Intervention: 69.05 (BL) 73.26 (10 weeks) Control: 72.29 (BL), 72.14 (10 weeks) *p*-value: 0.571	Not reported	Not reported	Not reported

Note: ICER: incremental cost-effectiveness ratio, QALY: quality-adjusted life year, RCT: randomized controlled trial, BL: baseline, HRQL: health-related quality of life. * Indicates statistically significant result.

## Data Availability

No new data were created or analyzed in this study. Data sharing is not applicable to this article.

## Supplementary Materials

The following supporting information can be downloaded at: https://www.mdpi.com/article/10.3390/curroncol30100642/s1, Table S1: Participant and intervention details.
