# Supplementary material for: Generic Health Utility Measures in Exercise Oncology: A Scoping Review and Future Directions
Source: Curr Oncol. 2023 Sep 28;30(10):8888–901. doi: 10.3390/curroncol30100642 (PMC10605555; doi:10.3390/curroncol30100642)
Supplement: Supplementary file 1 [file curroncol-30-00642-s001.zip › curroncol-2535688-supplementary.pdf]

Supplementary Table S1. Participant and intervention details.

Study/country	Cancer type N (%)	Groups	Mean Age (SD)	Sex n (%)	Timepoint** and duration of exercise intervention	Exercise prescription parameters				Control or comparison intervention
						Type	Intensity	Frequency/ Duration	Setting	
Gordon et al. 2017 [36], Australia	Breast 194 (100)	Full study: Face to face intervention: 67 Telephone intervention: 67 Control- 60<br><br>Included in CUA: Intervention- 134, Control- 60	Face to face- 51.2(8.8) Telephone- 52.2 (8.6) Control- 53.9 (7.7)	194 (100) female	After surgery. During the trial, 69% of women underwent chemotherapy, 71% underwent radiotherapy, and 64% began hormone therapy.<br><br>8 months	Aerobic interval and muscular strength training	Moderate	Supervised sessions weekly for months 1-2, biweekly for months 3-4, monthly for months 5-8. Unsupervised sessions were 2-4 times per week	Unsupervised and telephone sessions were home-based, supervised sessions for in-person group were clinic-based	Usual care
van Waart et al. 2018 [32], The Netherlands	Breast 153 (100)	Intervention: 76 Control: 77	Intervention: 49.9 (8.4) Control: 51.6 (8.8)	Intervention: 2 (3) male, 74 (97) female<br><br>Control: 77 (100) female	During treatment<br><br>Varied- each participant exercised for the duration of their chemotherapy regimen<br><br>Intervention group participants had mean of 110.8 (SD = 28.6) chemotherapy days	Aerobic and resistance training	Moderate to high	50 min sessions 2x/wk	Clinic-based, supervised	Usual care

Haines et al. 2010 [37], Australia	Breast 89 (100)	Interventio n: 46 Control: 43	Intervention: 55.9 (10.5) Control: 54.2 (11.5)	89 (100) female	During treatment 6 months	Strength, balance, shoulder mobility and cardiovascula r endurance program	Moderate	NR	Home-based unsupervise d	An active (sham intervention) control condition was employed consisting of flexibility and relaxation activities.
Ochi et al. 2022 [38], Japan	Breast 50 (100)	Interventio n: 25 Control: 25	Intervention- 48 (6) Control- 49 (5)	50 (100) female	After treatment 12 weeks	HIIT, personalized, body weight exercises delivered via a smartphone app	High	10 min sessions, 3x/wk	Home-based, unsupervise d	Control group received a smartwatch for 12 weeks
Cuesta-Vargas et al. 2014 [33], Spain	Breast 42 (100)	Interventio n: 22 Control: 20	Intervention: 47.3 (6.6) Control: 48.7 (9.7)	42 (100) female	After treatment 8 weeks	Deep water running, land-based mobility and strengthening exercise	Moderate-high	60 min sessions 3x/week	setting not reported, supervised	Usual care
Kampshoff et al. 2018 [29], The Netherlands	Overall n= 277 Breast 181 (65) Colon 49 (17) Lymphoma 26 (12) Ovarian 12 (4) Cervix 4 (1) Testis 5 (1)	High intensity: 139 Low to moderate intensity: 138	High intensity: 54 (10.7) Low to moderate intensity: 53 (11.4)	High intensity: 29 (21) male, 110 (79) female Low to moderate intensity: 26 (19) male, 112 (81) female	After treatment 12 weeks	Aerobic interval and muscular strength training	Low-moderate vs high	2x/wk; session duration NR	Clinic-based, supervised	Wait-list control (usual care)
May et al. 2017 [30], The Netherlands	Breast 204 (86)	Full study: intervention - 102, control-102 Included in CUA: intervention - 87, control- 78	Intervention- 50.0 (7.9), control- 49.4 (7.6)*	165 (100) female*	During treatment 18 weeks	Aerobic interval and muscular strength training	Moderate	1 hour sessions 2x/week	Clinic-based, supervised	Usual care

	Colon 33 (14)	Full study: intevention- 17, control- 16<br><br>Included in CUA: intervention - 14, control- 15	Intervention- 57.4 (11.2), control 59.1 (8.9)*	Intervention- 7 (50) male, 7 (50) female, control- 11 (73) male, 4 (27) female*						
van Dongen et al. 2019 [31], The Netherlands	Overall n= 109 Multiple myeloma 58 (53) non-Hodgkin lymphoma 51 (47)	Intervention: 54 Control: 55	Intervention: 52 (11) Control: 53 (12)	Intervention: 32 (59) male, 22 (41) female Control: 37 (67) male, 18 (33) female	After treatment<br><br>18 weeks	Aerobic interval and muscular strength training	High	60 min sessions, twice weekly for 1st 12 weeks, once weekly for last 6 weeks	Clinic-based, supervised	Usual care
Edmunds at al. 2020 [35], Australia	Prostate 100 (100)	Intervention: 50 Control: 50	Intervention: 71.9 (5.6) Control: 71.5 (7.2)	100 (100) male	After treatment<br><br>6 months	Aerobic and resistance training	Moderate-high	60 min sessions, 2x/week	Clinic-based, supervised	Control group: pedometer and a modified educational booklet with physical activity guidelines (150 min per week, moderate intensity)
Rosero et al. 2020 [34], Spain	Non-small-cell lung 34 (100)	Intervention: 21 Control: 13	Intervention: 74.5 (3.6) Control: 79.0 (3.0)	Intervention: 15 (79) male, 4 (21) female Control: 5 (71) male, 2 (29) female	During treatment<br><br>10 weeks	Aerobic, resistance, balance/coordination, and flexibility training	Moderate	45-50 min sessions, 2x/week	Research clinic, supervised	Usual care

*Participants included in the economic evaluation only
**Timepoint is in relation to cancer treatment
CUA- cost-utility analysis
